# Non-obstructed supracardiac total anomalous pulmonary venous connection with giant superior vena cava aneurysm

**DOI:** 10.1186/s12893-021-01135-0

**Published:** 2021-03-17

**Authors:** Yangbo Yan, Kai Yu, Yanlin Yang, Changping Gan

**Affiliations:** Department of Cardiovascular Surgery, West China Hospital, Sichuan University, No.37 Guo Xue Alley, Chengdu, 610041 China

**Keywords:** Total anomalous pulmonary venous connection, Superior vena cava, Aneurysm

## Abstract

**Background:**

Giant superior vena cava (SVC) aneurysm in non-obstructed supracardiac total anomalous pulmonary venous connection (TAPVC) is even more rare than in obstructed one, and this combination has not been reported.

**Case presentation:**

Here we reported a 29-year‐old young lady with non-obstructed TAPVC complicated with a giant SVC aneurysm. Routine TAPVC correction and tricuspid valve repair were done. Additionally, venoctomy was carried out to seek for its histological etiology and to avoid potential complications.

**Conclusions:**

If confirmed by further and larger experiences, for patients with non-obstructed supracardiac TAPVC with giant SVC aneurysm, surgical treatment of SVC aneurysm could be more aggressive to prevent cardiovascular complications.

## Background

Giant superior vena cava (SVC) aneurysm in obstructed supracardiac total anomalous pulmonary venous connection (TAPVC) is not a common congenital disease as reported [1–3]. A giant SVC aneurysm in non-obstructed TAPVC is more rare. Here we reported a 29-year‐old female patient with non-obstructied TAPVC complicated with a giant SVC aneurysm.

## Case presentation

**Fig. 1:**
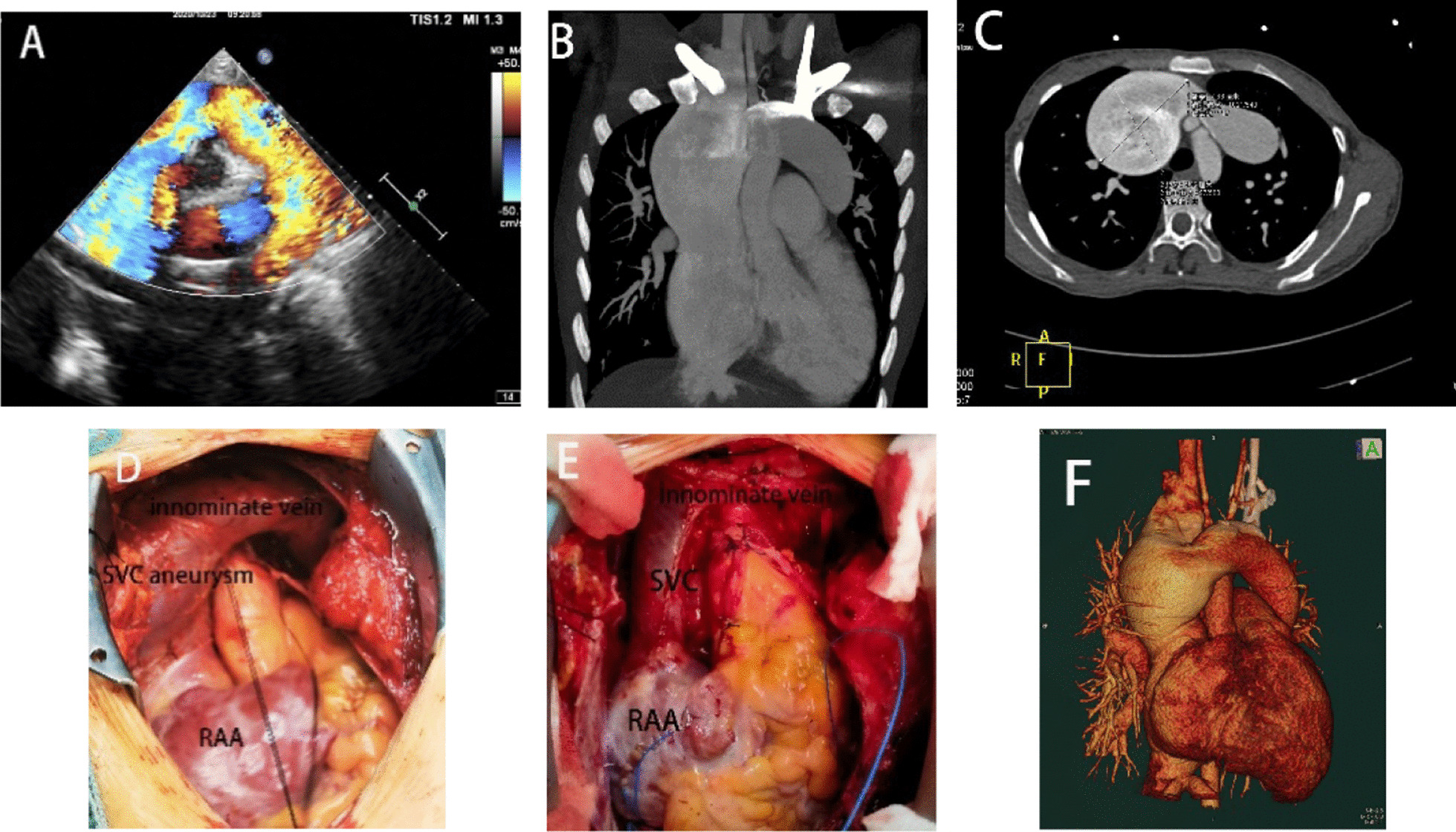
**a–b**; **f** Preoperative transthoracic echocardiography and CT showed supracardiac TAPVC with a giant venous arch. C CT showed the maximum diameter of the SVC. **d**–**e** Pre and postoperative view of SVC

We saw the patient in the clinic due to her dyspnea and impaired exercise tolerance. She looked emaciated and her Body Mass Index (BMI) was only 14.62 kg/m^2^. Her cardiac function was graded NYHAII according to the history. Echocardiography and computed tomography (CT) before the operation confirmed the diagnosis of supracardiac TAPVC with a giant venous arch, which were complicated with a 22 mm atrial septal defect (ASD) (Fig. 1a–b; f). The maximum diameter of the SVC was measured 7.68  × 6.32 cm (Fig. 1c). No obstruction throughout the venous pathway was noted neither by the echocardiography nor the CT. Angiography was conducted and the mean pulmonary artery pressure was measured as 22 mmHg with low pulmonary resistance. Intraoperative findings were consistent with all preoperative diagnosis (Fig. 1d).

To set up the cardiopulmonary bypass, along with the routine inferior vena cava(IVC)cannulation, two separate venous catheters were inserted into the right brachiocephalic vein and the innominate vein respectively. TAPVC correction was conducted by anastomosing the posterior wall of left atrium with the pulmonary venous confluence and ligating the vertical vein. The ASD was repaired with a bovine pericardial patch and a 30 mm plastic ring (MC3 4900, Edwards Life Science. Irvine, California, USA) was implanted to complete the tricuspid annular plasty. Considering that such a giant SVC aneurysm presented in a non-obstructed environment, we thought it might have its histological etiology and would take a higher risk of thrombosis and/or rupture. Therefore, a partial venoctomy was reasonable. With the distal veins controlled, the wall of the aneurysm was partially resected, and the diameter of SVC was reduced to a normal size (Fig. 1e). Pathology of the SVC revealed desmoplasia with hyaline degeneration and calcification in the vascular wall and mucoid degeneration in the focal area. Postoperative course was uncomplicated and she was discharged on the seventh postoperative day.

## Discussion and conclusion

Giant SVC aneurysm in supracardiac total anomalous pulmonary venous connection (TAPVC) is rare, a few cases in obstructed TAPVC were reported. [1–3] However, no report about non-obstructed TAPVC with SVC aneurysm was published so far. In the present case, no obstruction throughout the venous pathway was noted while the aneurysm was too large to neglect.

Some literatures have concluded that, compared with a saccular shape, a fusiform SVC aneurysm will usually have a benign prognosis, thus no aggressive treatment is needed [1–4]. However, for saccular aneurysm of SVC, surgical treatment is more recommended to avoid or to treat the complications. With median sternotomy and cardiopulmonary bypass, the aneurysm is totally resected in some publications [5–8]. While in other reports, the aneurysmatic sac is opened and removed, and then SVC is reconstructed with a pericardial patch [9, 10], or primarily sutured [11]. Thus, taking into consideration that the potentially histology-backgrounded aneurysm would increase the risk of cardiovascular complications, the angioplasty for SVC was done as above-mentioned. The result of pathology also implied her risk factors of cardiovascular diseases.

In spite of the insufficient literature in this area, the treatment for SVC aneurysm could be more aggressive to prevent cardiovascular complications in patients with non-obstructive supracardiac TAPVC with giant SVC aneurysm.

## Data Availability

Not applicable.

## Abbreviations

TAPVC: Total anomalous pulmonary venous connection
SVC: Superior vena cava
TVP: Tricuspid valvuloplasty
BMI: Body Mass Index
CT: Computed tomography
IVC: Inferior vena cava
ASD: Atrial septal defect
RAA: Right atrial appendage
